# Evaluation of an online intervention to promote hearing protective behavior in young people

**DOI:** 10.1016/j.invent.2026.100996

**Published:** 2026-09-01

**Authors:** Tjeerd Idger de Zeeuw, Gjalt-Jorn Peters, Lisanne de Regt, Marieke Pronk, Peter Verboon, Catherine A.W. Bolman

**Affiliations:** a Open University of the Netherlands, Heerlen, the Netherlands; b Department of Hearing Loss and Tinnitus Prevention, Dutch Consumer Safety Institute (VeiligheidNL), Amsterdam, the Netherlands

**Keywords:** Noise, Hearing protection, Ear- and headphone use, Hearing loss, Youth, Behavior change, Intervention

## Abstract

**Objective:**

The present study evaluated the effectiveness of a new online behavioral intervention (Listening type Check) as an extension to an online hearing test, to increase safe ear- and headphone use and change the underlying psychological factors in persons 16–21 years old.

**Design:**

An intensive longitudinal study with experimental design, using the experience sampling method, measured listening behavior and psychological factors two weeks before and after doing an online hearing test plus Listening type Check (intervention) or hearing test alone (control). Additionally, a process evaluation was performed.

**Study sample:**

Persons aged 16 to 21 years living in the Netherlands and using head- or earphones for at least one hour per week. Participants (*n* = 54) were on average 17 years old, 57% was male, and 90% theoretically educated.

**Results:**

Participants in the two weeks pre-intervention, used ear- or headphones for about 6 days a week, 2 h a day, mostly choosing a sound volume around 60% (on 0–100% scale). A loud volume (i.e. ≥60% when <18 years) was used for 40 min a day. These behaviors and underlying psychological factors did not change in the two weeks after doing the intervention.

**Conclusions:**

Participation in a new online behavioral intervention plus online hearing test did not increase safe ear- and headphone use or change underlying psychological factors. Participants were primarily theoretically educated and exhibited relatively safe listening behavior at baseline, which may explain the absence of an intervention effect. A new effect study with vocationally educated participants or other high-risk groups is recommended.

## Highlights

• A behavioral intervention to prevent hearing damage from headphones was evaluated.
• Participants (16–21 years) 4 weeks daily reported headphone use and beliefs.
• The intervention did not increase safe headphone use.
• The intervention did not change underlying psychological factors.
• Participants' safe headphone use at baseline may explain the findings.

## 1 Introduction

Most young people aged 12–25 years in Western countries use ear- or headphones for multiple hours a day (Dillard et al., 2022; de Zeeuw et al., 2026a). Although a safe sound level is chosen most of the time, the accumulation of loud volume moments over a week often exceeds the weekly maximum exposure level that is considered safe and puts a large portion of young people at risk of chronic tinnitus and noise-induced hearing loss (NIHL) (Lee and Kim, 2018; Ding et al., 2019). This risk is even greater when added to other situations in which young people are exposed to loud sounds, most notably during amplified music venues visits (Dillard et al., 2022; de Zeeuw et al., 2026a).

Hearing loss and tinnitus may have far-reaching consequences for a person's life, such as social isolation, increased depressive symptoms (Nordvik et al., 2018; Shukla et al., 2020; Li et al., 2014), and limited educational and employment opportunities (Emmett and Francis, 2015; Shan et al., 2020).

The risk for NIHL and tinnitus can be mitigated by reducing exposure to noise. In the context of ear- or headphones use, this can be accomplished by choosing a safe sound volume or using a volume limiter. High volumes are best avoided, but if chosen, it should be infrequent and for short durations. However, a recent study shows they are performed by a minority of young people (de Zeeuw et al., 2026a).

To increase safe ear- and headphone use among young people aged 12–25 years, the online behavioral intervention *Luistertypecheck* (“Listening type check”, hereinafter abbreviated as “LC”) was developed, as the group of young people potentially at risk to suffer hearing damage due to their ear- and headphone use is large, yet no systematically designed interventions exist to change these behaviors. Interventions to increase hearing protective behavior (HPB) in young people predominantly target earplug use and the main or only behavior change method used is information provision (Loughran et al., 2020; Khan et al., 2018; Bramati et al., 2024), which has low behavior change potential (Loughran et al., 2020; Khan et al., 2018; Bramati et al., 2024; ten Hoor et al., 2026).

### 1.1 Intervention development

The LC was developed using a systematic, evidence- and theory-based process informed by a systematic review (de Zeeuw et al., 2025), a qualitative study (de Zeeuw et al., 2026b), and a cross-sectional survey (de Zeeuw et al., 2026a). These studies identified key modifiable psychological determinants of HPB, which were translated into intervention content using Acyclic Behavior Change Diagrams (ABCDs) (Metz et al., 2022). ABCDs explicitly link determinants, behavior change principles, practical applications and target behaviors. Six determinants were targeted: experiential attitude (the degree to which a loud volume is perceived as pleasant), awareness (recognition of one's own listening behavior and hearing damage risk), perceived barriers (perceived obstacles to listening safely), capacity (perceived ability and confidence to perform safe listening behaviors), habit (the extent to which unsafe listening occurs automatically), and perceived threat susceptibility (perceived personal likelihood of developing NIHL or tinnitus). Determinant definitions are available in the repository of the Decentralized Construct Taxonomy (DCT) developed before the start of the study, accessible via https://psycore.one (Peters and Crutzen, 2024).

The intervention aimed to increase awareness, capacity and perceived threat susceptibility, weaken positive experiential attitudes towards loud listening, reduce perceived barriers and disrupt habitual unsafe listening.

Rather than targeting safe listening as one general behavior, the LC focused on four specific sub-behaviors that together determine cumulative sound exposure during personal listening device use: (1) choosing a safe volume, because sound intensity is the primary determinant of noise exposure; (2) enabling the device's volume limiter, which prevents accidental or habitual increases to hazardous sound levels; (3) lowering volume when the device warns for excessive exposure, thereby responding immediately to potentially harmful listening levels; (4) using ear- or headphones that sufficiently attenuate environmental noise, reducing the need to compensate for background noise by increasing volume.

Safe listening thresholds differed by age: listening above 60% of maximum volume was considered potentially unsafe for participants <18y; for ≥18y the threshold was two-thirds (≈67%). These thresholds broadly correspond to WHO-recommended age-specific maximum acceptable weekly noise exposure levels of 75 dB(A) and 80 dB(A) (40-h LAeq), respectively (World Health Organization, 2019).

Overall risk of hearing damage was determined using two algorithms. The first algorithm estimated weekly equivalent continuous sound level (LAeq) –average sound energy over a defined period- by combining self-reported listening frequency (D, days per week), duration (H, hours per day) and sound level (L, volume percentage converted to dB(A)): LAeq = L - 10*log(8/H) - 10*log*(5/D).* Sound level (dB(A)) was estimated using 0.6322 * volume percentage + 38.78, a slightly adapted version of the equation used by Paping, Vroegop (Paping et al., 2022), with a somewhat steeper slope reflecting more recent measurements of contemporary smartphone–earphone/headphone combinations. LAeq values above 80 dB(A) (≥18y) and 75 dB(A) (<18y) were classified as ‘increased risk’, in accordance with WHO-criteria (World Health Organization, 2019).

The second algorithm classified participants as ‘increased risk’ when cumulative weekly listening above the age-specific loud-volume threshold exceeded 1.5 h (<18y) or 2.5 h (≥18y), assuming loud-volume levels of ∼89 dBA (80% volume) and ∼ 92 dBA (85% volume), respectively. Participants exceeding either algorithm's criterion were classified as being at increased risk of hearing damage.

After briefing, a creative and a web design agency built the LC. The ABCDs informed the creative agency which psychological factors to target, and how. The briefing contained two key conditions: the intervention should extend the online “Earcheck” (“EC”), a validated online hearing test that detects hearing loss in people 12–25 years of age (https://hoortest.oorcheck.nl), and it should specifically match vocationally educated young persons, as this group is most at risk for NIHL due to noise overexposure (de Zeeuw et al., 2026a; Van der Molen et al., 2018).

The complete intervention development process, including matrices of change objectives, ABCDs, and operationalizations, has been made publicly available to facilitate transparency and replication (https://osf.io/suy54).

### 1.2 Study aims

The present study aimed to evaluate the effectiveness of the EC plus LC to increase safe ear- and headphone use, intention to use ear- and headphone safely, and change underlying psychological factors, compared to EC alone. Additionally, a process evaluation was performed.

## 2 Methods

### 2.1 Protocol and repository

The study's protocol, including questionnaires, and all other materials, are publicly available at the study's repository at https://osf.io/suy54.

### 2.2 Study participants

Persons 16 to 21 years of age that lived in the Netherlands and used ear- or headphones for at least one hour per week.

### 2.3 Study design, procedure and randomization

An intensive longitudinal experimental study, using the experience sampling method (ESM), measured participants' listening behavior and underlying psychological factors two weeks before and two weeks after participation in either EC plus LC (intervention) or EC alone (control). Additionally, a process evaluation of LC and EC was performed.

First, participants completed an online survey, via LimeSurvey (Limesurvey GmbH, 2025), measuring background variables, listening behavior, and intention to listen safely. Participants that completed the survey were randomly assigned by LimeSurvey to the intervention or control condition for the next four weeks.

Next, participants downloaded the LifeData ESM application (LifeData Corp., Marion, IN) and the study's survey package to their smartphone. Participants received daily questions on the application for 28 days, measuring listening behavior, intention to listen safely, and psychological factors.

After 14 days, participants took part in either the EC plus LC or EC alone and subsequently completed the process evaluation survey about the LC and/or EC via LimeSurvey. Next, participants continued answering daily questions via the application for another 14 days.

### 2.4 Recruitment

Participants were recruited via high schools and institutions of intermediate vocational education. Teachers and staff members brought the study to students' attention, and distributed a digital leaflet mentioning that completing the 28-day participation period was rewarded with a €50 gift voucher and the chance to win one of ten pairs of noise-canceling headphones.

### 2.5 Informed consent

Participants signed an informed consent form after reading a digital information letter about the study and potential implications of participation.

### 2.6 Measurements

The baseline and process evaluation survey items were presented once. Items prompted via the app, were presented daily and every third day at 8 pm (to minimize interference with school or work), and could be answered until 11.30 pm.

The exact operationalization of each variable measured in the study can be found in the study's protocol (https://osf.io/suy54).

The *baseline survey* measured gender, age, educational level, hearing problems, ear- and headphone use (days/week, hours/day, most frequent volume, time at loud volume), and presence and use of a volume limiter and/or warning function for excessive volume on mobile phone.

The *process evaluation survey* contained 5-point, 10-point, and open questions measuring participants' experience with the LC and/or EC.

*Daily ESM-items* assessed duration of ear- and headphone use, usual listening volume, and duration of loud-volume listening. Loud listening was defined as >60% of maximum volume for participants <18y and >two-thirds for participants ≥18y.

Items presented *every third day* via *the ESM app* (in varying composition) assessed the use of a volume limiter or volume warning function (one of the two, depending on the participant's device), intention to use one of these functions, intention to choose a safe volume, and psychological factors underlying safe listening.

The measurement of the six psychological factors targeted in the LC was guided by the DCT (Peters and Crutzen, 2024), and assessed similarly as in a previous study by the authors (de Zeeuw et al., 2026a). As psychological factors do not change quickly, they were measured every third day, mostly with one item; two items were used if distinct subfactors were involved. Long questionnaires potentially lead to more non-response, lower data quality, and higher dropout (Eisele et al., 2020; Galesic and Bosnjak, 2009; Revilla and Ochoa, 2017). Brief measures have shown sufficient convergent validity (Crutzen and Peters, 2023).

### 2.7 Sample size

As formal sample size guidelines for repeated *N* = 1 intervention studies are lacking, we aimed to include at least 15 participants per condition based on the planned number of repeated measurements (28 daily and 9–10 every-third-day observations) (Myin-Germeys and Kuppens, 2021). Allowing for 50% attrition, 30 participants per condition were recruited.

### 2.8 Participant attrition and exclusion

Answering questions daily for 28 days is demanding and may lead to significant attrition especially with adolescent and young adult participants. To reduce attrition, we kept the number of daily items limited (<2 min completion time), and provided incentives as recommended by van Roekel et al. (2019). Participants answering at least 75% of the daily pre- and post-intervention questions and completing the baseline and post-intervention surveys received a €50 gift card and the chance to win a pair of headphones.

ESM studies report an average compliance of about 80% (Wen et al., 2017; Williams et al., 2021). Therefore, we excluded participants with <75% compliance in responding the daily questions.

### 2.9 Analyses

Data points more than three standard deviations higher or lower than the mean were visually inspected. Two variables contained data points considered outliers that were winsorized: values greater than 780 min for Headphone Use (in minutes/day) were replaced by a value of 780 min, and values greater than 600 min for Loud Sound Volume (in minutes/day) were replaced by a value of 600 min. This occurred, four and two times, respectively.

For baseline and process evaluation survey items, responses were mandatory and therefore held no missing values. Participants with >25% missing data on an ESM-app item were excluded from the analyses of the particular variable (>7 missings for daily items; >3 for items presented every third day).

New variables were created for education (vocational vs theoretical) and for weekly hours of loud-volume listening. “Vocational education” included secondary vocational education (mbo), pre-vocational secondary education (vmbo), practical education, and primary school; however, all vocationally educated participants were enrolled in secondary vocational education (mbo). “Theoretical education” comprised senior general secondary education (havo), pre-university education (vwo), higher professional education (hbo), and university. Weekly hours of loud-volume listening was created by multiplying the hours of loud volume a day with the weekly days of ear- and/or headphone use. Items for psychological constructs measured with more than one item were analyzed individually and averaged.

Following the inspection and preparation of the data, descriptive statistics were produced for cross-sectional baseline and process evaluation data, and longitudinal pre- and post-intervention measurements for the total sample (*N* = 54), intervention (*n* = 26) and control (*n* = 28) group. For continuous variables, the mean with 95% confidence interval (CI) was reported. For categorical variables, number and relative frequency was reported. Estimates with CIs that overlap were considered to be not significantly different from each other.

Generalized logistic models were planned to visualize trajectories of change in behavior and/or psychological factors. If descriptive statistics showed no signs of changes after participation in the intervention, these analyses would be omitted.

Inspection and preparation of the data and descriptive analyses were conducted with IBM SPSS statistical software, version 25 (*IBM Corp.*, 2017). For the analyses of repeated *N* = 1 measurements, R version 4.1.0 (R Core Team, 2022), specifically the “scda” package for generalized logistic models (Verboon and Peters, 2020), would be used.

### 2.10 The intervention

#### 2.10.1 Development

After extensive briefing, creative agencies presented intervention proposals from which “the listening type” was selected by the project's planning group, consisting of health behavior researchers and hearing protection specialists, and a group of seven vocational education students. The selected agency then further elaborated their idea and integrated behavior change elements in co-creation with vocational education students. Tweaks were made based on feedback from researchers and planning group. The final concept intervention was then technically built by an IT-design agency.

#### 2.10.2 Features

The LC is a digital, online tool for young people (12–25 years) to: 1) discover their ‘listening type’ (which is the tool's fun element), 2) determine if their listening behavior is safe, 3) receive personalized tips and advice for safe listening. The LC is lined up right after the EC, although it could potentially also be used as stand-alone.

**Fig. 1:**
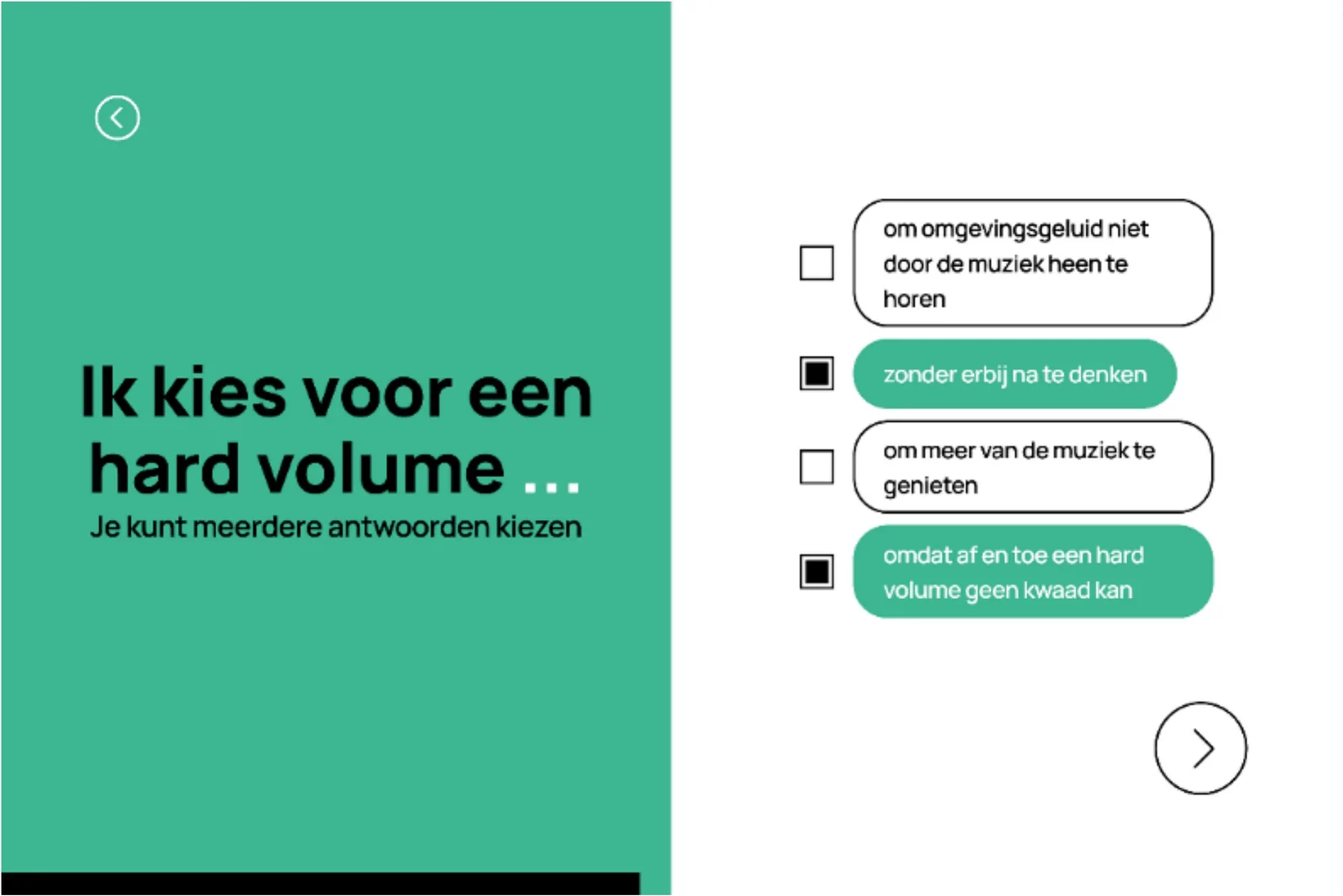
Questionnaire item from the LC. Here: “I choose a loud volume...” (multiple answers possible).

Participants first completed items determining their listening type, followed by questions on listening behavior (used to classify their risk as ´increased´ or not), their interpretation of temporary tinnitus, and items assessing reasons to choose a loud volume (see Fig. 1). Tailored feedback then provided personalized listening advice, practical information, an individualized hearing damage-risk estimate with interactive exposure sliders, and an optional habit-change plan with weekly reminder emails.

**Fig. 2:**
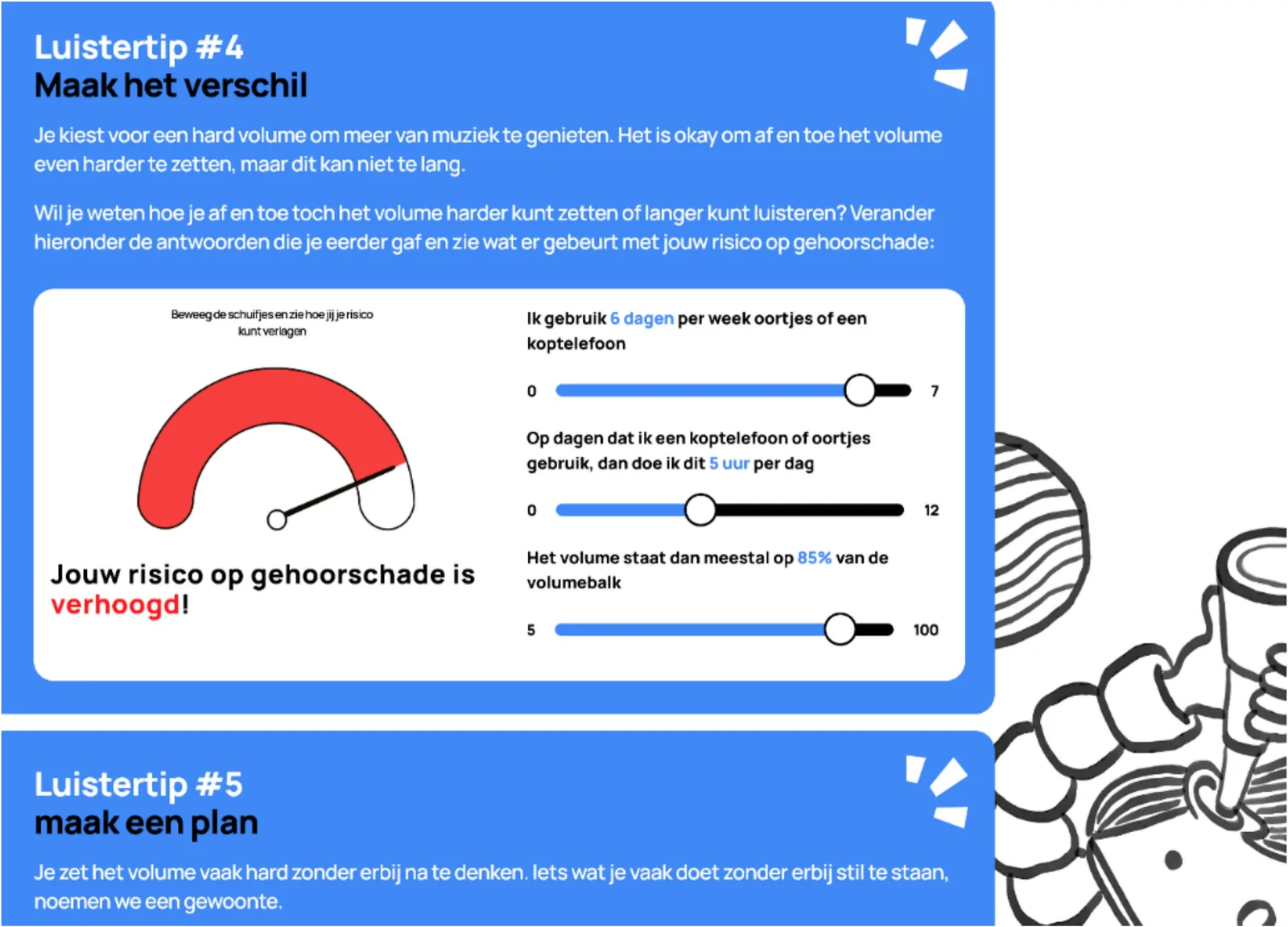
A listening tip from the LC. Move the sliders for days, hours, and volume and see how you can reduce your hearing damage risk in a way that works for you.

The results were presented in a lay-out and formulation matching the participant's listening type. First, the listening type was described. Next, tailored to earlier answers on ear- and headphone use, practical information was provided about using a volume limiter and choosing safe ear- or headphones models (promoting capacity). Each participant was also shown a short transcribed interview, in which a doctor discusses tinnitus and advised safe listening (promoting threat susceptibility), and listened to audio fragments illustrating how hearing loss and tinnitus may sound. Next, based on the reported listening behavior, a participant's estimated hearing damage risk was shown (promoting awareness). Using switches for days, hours, and volume, participants could instantly see how changes affected their risk (promoting capacity, see Fig. 2). Finally, participants could set up a habit-change plan with a time line to gradually adjust their listening behavior, supported by weekly reminder emails, containing each week's plan.

## 3 Results

### 3.1 Sample characteristics

Of the participants that completed the baseline survey and subsequently started participation via the ESM app (*n* = 68), 14 participants were excluded because they either did not complete the intervention and/or post-intervention survey (*n* = 12), or responded to an insufficient number of ESM-questions (*n* = 2), resulting in 54 participants. Table 1 presents the intervention and control group's characteristics at baseline and listening behavior before and after the intervention.

**Table 1:** Demographics and listening behavior of the total sample, intervention group, and control group.

Variable †	Total sample (*N* = 54)	Intervention group (*n* = 26, 48.1%)	Control group (*n* = 28, 51.9%)
Age	17.11 (16.72–17.50)	16.81 (16.36–17.25)	17.39 (16.75–18.04)
Gender			
Male	31 (57.4%)	15 (57.7%)	16 (57.1%)
Female	23 (42.6%)	11 (42.3%)	12 (42.9%)
Education			
Theoretically educated	50 (92.6%)	24 (92.3%)	26 (92.9%)
Vocationally educated	4 (7.4%)	2 (7.7%)	2 (7.1%)
Listening behavior assessed via online survey (cross-sectionally measured at baseline)			
Days/week headphone use	5.41 (4.95–5.87)	5.54 (4.92–6.16)	5.29 (4.58–5.99)
Hours/day of headphone use	2.58 (2.11–3.06)	2.56 (1.90–3.21)	2.61 (1.88–3.33)
Sound volume (0–100%) most of the time	60.93 (55.61–66.24)	60.19 (52.52–67.87)	61.61 (53.76–69.45)
Hours/day loud sound volume‡	1.06 (0.79–1.34)	1.13 (0.68–1.59)	1.00 (0.64–1.36)
Listening behavior assessed via app – Overall (averages of 28 consecutive daily measurements)			
Days/week headphone use	5.91 (5.65–6.17)	6.29 (5.92–6.65)	5.56 (5.19–5.92)
Hours/day of headphone use	2.21 (2.08–2.33)	2.06 (1.89–2.23)	2.37 (2.18–2.55)
Sound volume (0–100%) most of the time	56.29 (54.93–57.65)	54.15 (52.32–55.99)^a^	58.56 (56.58–60.55)
Minutes/day loud sound volume	38.45 (34.33–42.57)	32.33 (26.89–37.76)^a^	44.99 (38.79–51.19)
Listening behavior assessed via app – Pre-intervention (averages of 14 consecutive daily measurements via app)			
Days/week headphone use	6.17 (5.82–6.53)	6.50 (5.99–7.00)	5.88 (5.37–6.38)
Hours/day of headphone use	2.01 (1.86–2.17)^b^	1.84 (1.62–2.06)	2.19 (1.98–2.40)
Sound volume (0–100%) most of the time	56.44 (54.52–58.36)	54.00 (51.39–56.61)	58.86 (56.05–61.66)
Minutes/day loud sound volume	41.05 (35.14–46.97)	29.15 (21.93–36.38)^a^	52.95 (43.80–62.11)^b^
Used volume limiter today (from 1 to 5, “never” – “always”)	4.01 (3.68–4.34)	4.02 (3.58–4.46)	4.00 (3.46–4.54)
Listening behavior assessed via app – Post-intervention (averages of 14 consecutive daily measurements via app)			
Days/week headphone use	5.65 (5.23–6.02)	6.08 (5.56–6.60)	5.21 (4.69–5.74)
Hours/day of headphone use	2.43 (2.23–2.63)	2.29 (2.04–2.55)	2.58 (2.26–2.90)
Sound volume (0–100%) most of the time	56.13 (54.22–58.03)	54.31 (51.71–56.91)	58.21 (55.40–61.01)
Minutes/day loud sound volume	35.54 (29.83–41.25)	35.65 (27.45–43.85)	35.42 (27.48–43.37)^b^
Used volume limiter today (from 1 to 5, “never” – “always”)	4.06 (3.70–4.43)	3.93 (3.48–4.38)	4.35 (3.70–4.99)

a 95% confidence intervals of intervention and control group diverge.

b 95% confidence intervals of pre- and post-intervention period diverge.

† Mean and 95% confidence interval are displayed for all variables, except for the variables gender and education, which display number (*n*) and percentage (%).

‡ Loud sound volume:>two-thirds of the volume bar is loud for participants aged ≥18y, and >60% is loud for those <18y.

More than 90% of study participants was theoretically educated. Visual inspection revealed no differences between intervention and control group in demographic background or listening behavior, except for two variables. First, participant's most chosen sound volume, as assessed via the app for 28 days, is higher for the control than intervention group. Second, in the 14 days before the intervention, the control group listens more at a loud sound volume than the intervention group.

### 3.2 Intervention effects

#### 3.2.1 Changes in listening behavior

**Fig. 3:**
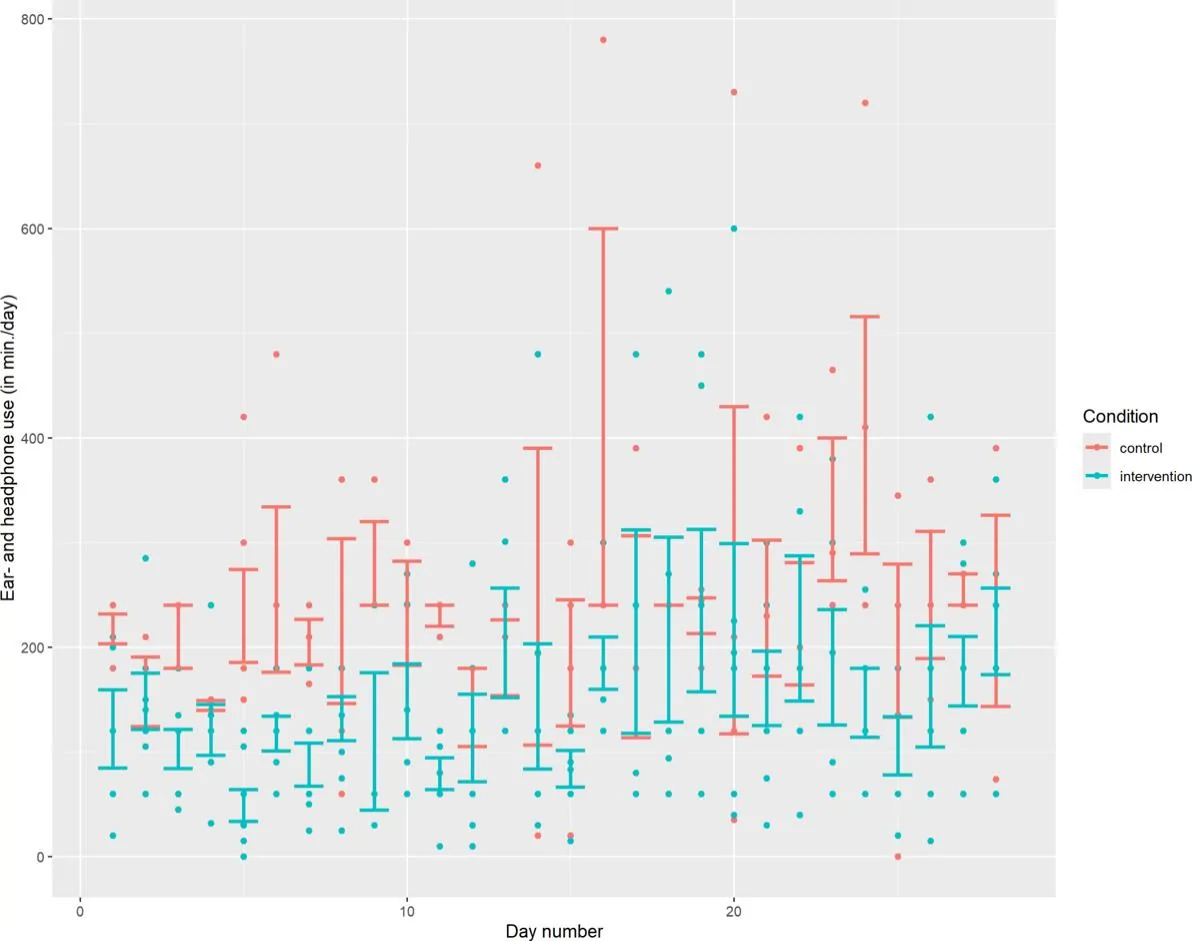
Ear- and headphone use (in minutes/day) during 28 days of daily measurements via ESM-app. On day 14 (halfway between the first and last day of measurements), participants participated in the EC plus LC (intervention; green), or the EC (control; orange).

**Fig. 4:**
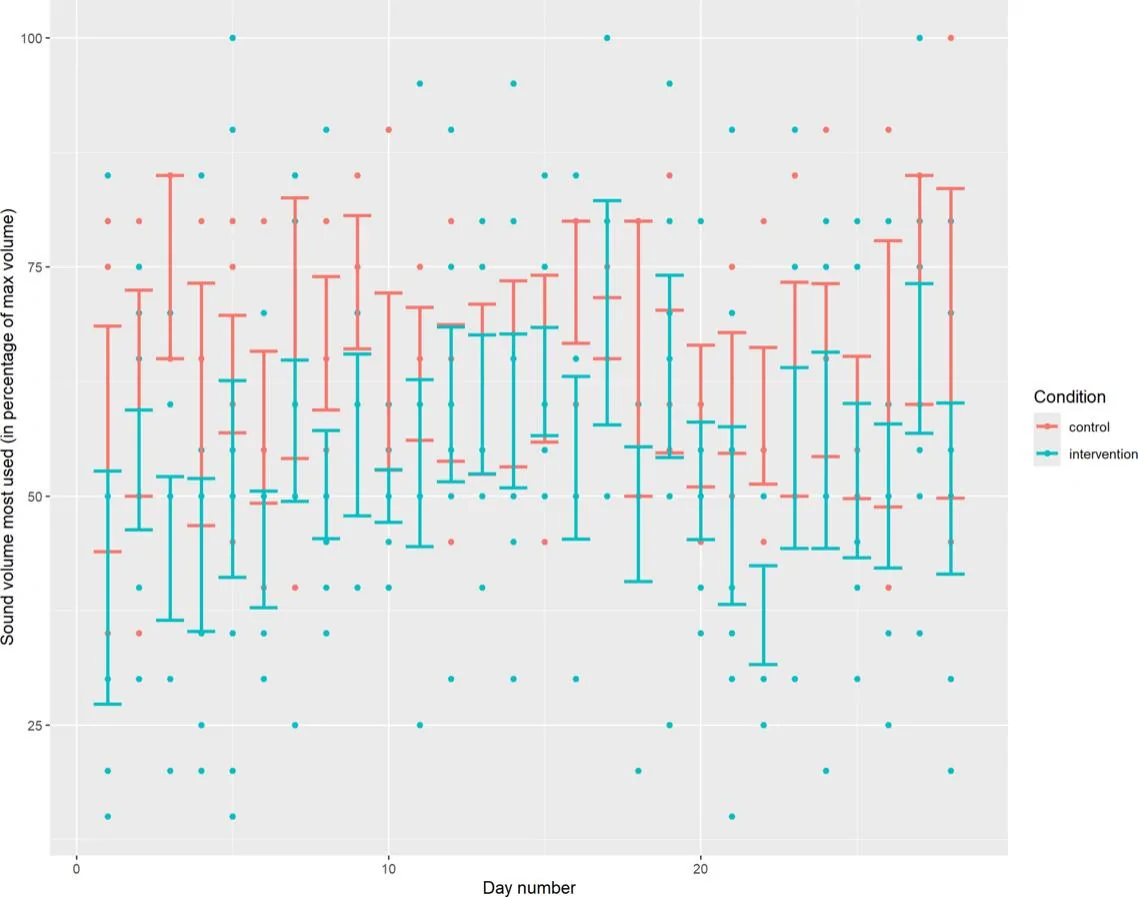
Sound volume mostly used (in percentage of maximum volume) during 28 days of daily measurements via ESM-app. On day 14 (halfway between the first and last day of measurements), participants participated in the EC plus LC (intervention; green), or the EC (control; orange).

**Fig. 5:**
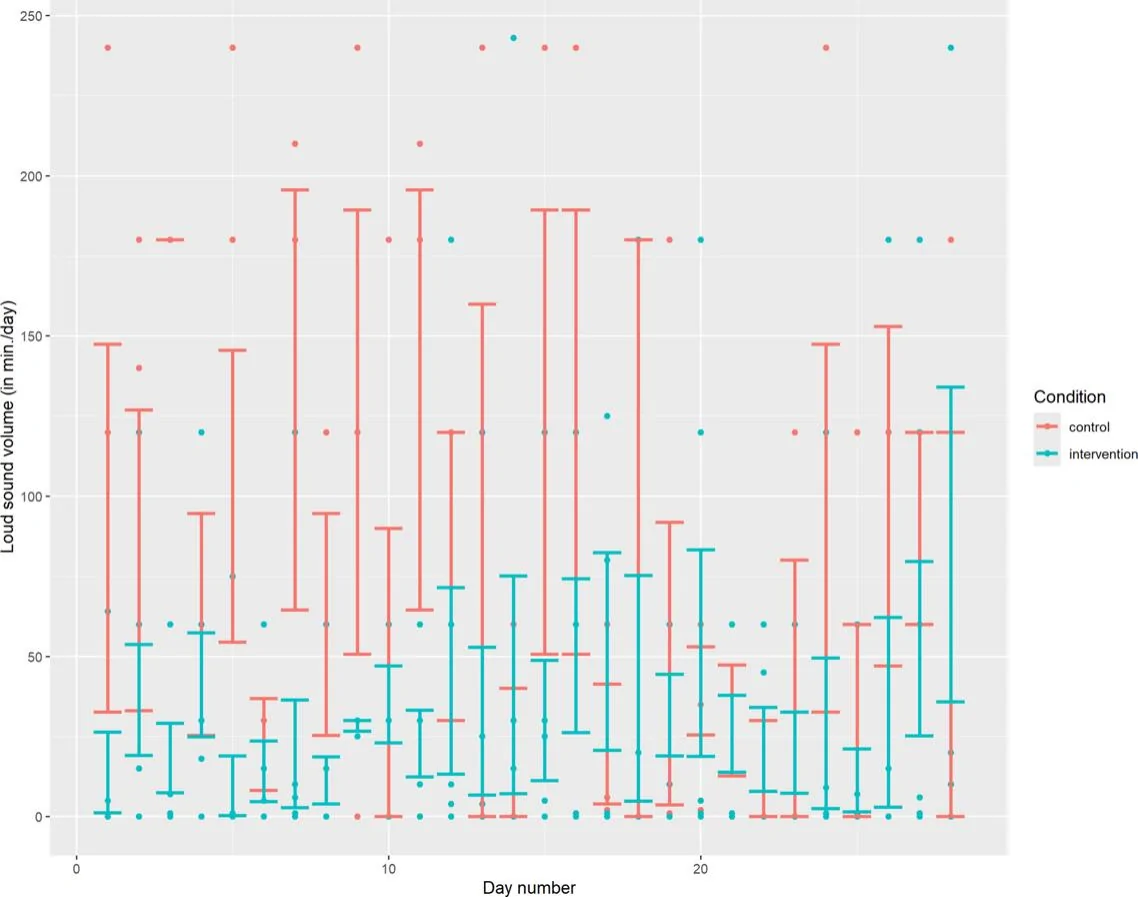
The use of a loud sound volume (in minutes/day) during 28 days of daily measurements via ESM-app. On day 14 (halfway between the first and last day of measurements), participants participated in the EC plus LC (intervention; green), or the EC (control; orange).

The only difference between pre- and post-intervention that can be observed in Table 1 is a reduction of loud sound volume listening in the control group. Fig. 3 illustrates that the minutes a day of ear- and headphone use, overall did not change in the 14 days post-intervention compared to the 14 days pre-intervention. The same continuation of listening patterns applies to the sound volume most chosen when using ear- or headphones, as shown in Fig. 4. In line with the data in Table 1, Fig. 5 illustrates that pre-intervention, participants in the control group overall listened more minutes a day at a loud sound volume than participants in the intervention group. Post-intervention, due to a decline in the control group, the use of a loud sound volume is equal for control and intervention group, at 35 min a day. In the intervention group, overall, the use of a loud sound volume did not decline after the intervention.

#### 3.2.2 Changes in psychological factors

Table 2 shows that participation in the LC and/or EC is not followed by a change in psychological factors: mean scores for the period before and after doing the LC and/or EC have overlapping CIs for both the intervention and control group.

**Table 2:** Relevant psychological factors before and after participation in LC and/or EC.

Variable†, ‡	Before or after LC and/or EC	Total sample (*N* = 54)	Intervention group (*n* = 26)	Control group (*n* = 28)
Intention to choose a safe volume¥ (from 1 to 5, “definitely not” to “absolutely”)	Before¤	3.78 (3.62–3.95)	3.87 (3.66–4.07)	3.70 (3.45–3.96)
	After	3.85 (3.69–4.02)	3.93 (3.72–4.14)	3.79 (3.54–4.04)
Intention to use volume limiter (from 1 to 5, “definitely not” to “absolutely”)	Before	4.29 (4.10–4.48)	4.39 (4.16–4.61)	4.15 (3.81–4.48)
	After	4.22 (4.00–4.45)	4.21 (3.95–4.48)	4.23 (3.80–4.66)
Intention to lower volume after warning (from 1 to 5, “definitely not” to “absolutely”)	Before	3.96 (3.64–4.27)	3.50 (2.95–4.05)	4.11 (3.74–4.49)
	After	4.36 (4.13–4.59)	4.00 (3.62–4.38)	4.51 (4.23–4.79)
Awareness (from 1 to 5, “definitely not” to “absolutely”)	Before	3.28 (3.04–3.52)	3.48 (3.18–3.79)	3.07 (2.71–3.44)
	After	3.60 (3.38–3.83)	3.70 (3.41–4.00)	3.49 (3.14–3.85)
Barriers (from 1 to 5, “definitely not” to “absolutely”)	Before	2.42 (2.19–2.65)	2.43 (2.10–2.75)	2.42 (2.08–2.75)
	After	2.51 (2.27–2.76)	2.42 (2.11–2.73)	2.61 (2.22–3.00)
Capacity (from 1 to 5, “definitely not” to “absolutely”)	Before	3.88 (3.72–4.05)	4.02 (3.80–4.24)	3.77 (3.53–4.01)
	After	3.90 (3.74–4.07)	3.96 (3.74–4.18)	3.85 (3.60–4.09)
Experiential Attitude – Joy (from 0%–100%, “minimum” to “maximum”)	Before	67.37 (65.08–69.65)	66.52 (63.67–69.36)	68.14 (64.58–71.69)
	After	67.37 (64.96–69.78)	66.79 (63.54–70.05)	67.97 (64.35–71.58)
Experiential Attitude – Energy (from 0%–100, “minimum” to “maximum”)	Before	70.26 (68.03–72.49)	70.75 (67.91–73.58)	69.79 (66.32–73.26)
	After	68.57 (66.21–70.94)	66.86 (63.75–69.98)	70.17 (66.63–73.71)
Habit (from 1 to 5, “never” to “always”)	Before	3.64 (3.49–3.84)	3.76 (3.51–4.02)	3.53 (3.22–3.82)
	After	3.49 (3.28–3.71)	3.47 (3.19–3.75)	3.52 (3.19–3.86)
Threat susceptibility, hearing (from 1 to 5, “very small” to “very large”)	Before	3.59 (3.45–3.76)	3.80 (3.62–3.98)	3.40 (3.18–3.62)
	After	3.62 (3.47–3.77)	3.83 (3.64–4.02)	3.41 (3.16–3.65)
Threat susceptibility, tinnitus (from 1 to 5, “very small” to “very large”)	Before	3.65 (3.50–3.81)	3.78 (3.59–3.96)	3.53 (3.29–3.77)
	After	3.68 (3.52–3.84)	3.77 (3.58–3.97)	3.59 (3.35–3.83)

† Mean and 95% confidence interval are displayed for all variables.

‡ The N at top of table are maximum values and may differ, depending on relevance of variable to a participant and missing values.

¥ Safe volume: ≤67% of the volume bar for participants ≥18y, and ≤60% for participants <18y.

¤ “Before” and “after” refer to the period *before* and *after* participation in the LC and/or EC, respectively.

As no relevant changes were discerned after doing the LC and/or EC, no generalized logistic analyses were performed to visualize patterns of change.

### 3.3 Process evaluation

Table 3 shows that the EC and LC were generally positively evaluated, with similar evaluations of the EC and LC. However, participants evaluated the capacity of the EC and LC to promote hearing protective behavior as limited (about 2.5). Overall, the EC was evaluated with a 7.4 and the LC with a 7.2.

**Table 3:** Process evaluation of the EC and LC.

Variable†	EC (Total sample; *N* = 54)	LC (Intervention; *n* = 26)
Evaluation EC and LC		
How much did you enjoy doing the EC/LC? (from 1 to 5, “did not enjoy it at all” to “enjoyed it a lot”)	3.33 (3.09–3.56)	3.50 (3.19–3.81)
How clear did you find the EC/LC? (from 1 to 5, “not at all clear” to “very clear”)	4.00 (3.74–4.26)	3.93 (3.51–4.35)
Is the EC/LC intended for young people like you? (from 1 to 5, “definitely not” to “definitely yes”)	4.04 (3.82–4.26)	4.07 (3.72–4.42)
Will young people protect their hearing better after doing the EC/LC? (from 1 to 5, “definitely not” to “definitely yes”)	2.55 (2.29–2.80)	2.36 (1.95–2.77)
What final grade do you give the EC/LC? (1–10)	7.38 (7.08–7.68)	7.21 (6.63–7.80)

† Mean and 95% confidence interval are displayed for all variables.

## 4 Discussion

A possible explanation for the absence of an intervention effect in the present study is the limited room for improvement. Pre-intervention, the sound volume most commonly chosen by study participants was around 60% of maximum volume, and a loud volume was used for roughly 40 to 60 min on days of headphone use. Added up over a week, the latter amounts to an average of 3.5 to 5.5 weekly hours. Although this exceeds safe noise exposure,^1^ it is considerably less than observed elsewhere: in a recent study the number of weekly hours at loud volume was 1.3 to 2 times higher for theoretically educated participants, and 2.3 to 3.7 times higher for vocationally educated participants (de Zeeuw et al., 2026a).^2^

As previous research (de Zeeuw et al., 2026a) shows, vocationally educated adolescents and young adults, in comparison to theoretically educated peers, engage less in safe listening behavior. In particular, those vocationally educated from 12 to 18 years are at risk: nearly three-quarters of them are overexposed to noise due to their ear- and headphone use (de Zeeuw et al., 2026a).

In the present study, less than 1 out of 10 participants was vocationally educated. The underrepresentation of vocationally educated participants limits interpretation of the findings. This group generally shows riskier listening behavior (de Zeeuw et al., 2026a), suggesting greater potential for behavior change than observed in the present sample. Consequently, both intervention effectiveness and acceptability remain uncertain for the primary target population.

Another possible explanation for the absence of an intervention effect is the perhaps incomplete application of the ABCDs developed to guide the intervention process. In the final version of the LC, these ABCDs have been applied to a limited extent.

Furthermore, changing entrenched listening habits may require a more intensive and longer-lasting intervention than the approximately 15-min LC (Verplanken and Orbell, 2022), despite the optional weekly action plans and reminder emails.

The absence of an intervention effect leads to three recommendations, following sequential steps. First, to assess the effect and acceptance of the LC in vocationally educated participants and other high-risk groups, a new evaluation study including these groups should be conducted. Second, if changes are absent or limited, the intervention can be expanded with more and/or more elaborated behavior change principles, as drawn up earlier in ABCDs. Third, if the expanded LC-intervention remains unable to increase safe ear- and headphone use, a switch to a longer-term intervention with a more intensive and integral character (e.g. offered within a school context) should be considered.

A recommendation for future hearing protection research is to include vocationally educated participants, especially those aged 12–18 years, as they appear to be most overexposed to ear- and headphone noise (de Zeeuw et al., 2026a). For the current study, we chose not to include participants aged 15 years and younger to avoid the need for active parental consent, which in previous studies proved to be time-consuming. However, this had the undesirable result that no pre-vocational secondary education students participated in the study, as these students are mainly younger than 16 years.

The study's process evaluation showed generally positive outcomes. The intervention scored satisfactorily on all points except one: participants evaluated the intervention's ability to change behavior as limited. Possibly the urgency to change is perceived as low when behavior is relatively safe. Replicating the study with participants that exhibit more risky listening behavior can reveal whether this changes this perception.

### 4.1 Limitations

Less than 10% of study participants were vocationally educated, and those aged 12 t0 15 years were absent. Therefore, the LC-intervention could not be evaluated in the group that is most at risk to incur hearing damage from ear- and headphone use (de Zeeuw et al., 2026a).

### 4.2 Strengths

The systematic development of the intervention, using ABCDs, enabled to measure change in precisely those behaviors and psychological factors targeted in the intervention. Four weeks of daily measurements plus a baseline survey resulted in the collection of a large set of data per participant over a longer period, which strengthens the reliability of the findings.

### 4.3 Conclusion

The intervention under study did not result in a change in safe ear- and headphone use or underlying psychological factors. Participants were primarily theoretically educated and exhibited relatively safe listening behavior at baseline, which may explain why no intervention effect was observed. A new study with vocationally educated participants or other high-risk groups is recommended.

## Ethical statement

This study has been granted ethical approval by the ethics committee of the Faculty of Psychology of the Open University, reference number U202406529.

## Declaration of competing interest

The authors report there are no competing interests to declare.

## Data Availability

Materials are publicly available at the study's Open Science Framework (OSF) repository at https://osf.io/suy54.

## Footnotes

1 In de Zeeuw et al. (2026a) safe noise exposure was defined as ≤2.5 h a week at ≥70% of maximum volume when using earphones, headphones or speakers.

2 The greater exposure to a loud volume was observed in spite of the higher threshold applied for “loud volume” in that study: ≥70% of maximum volume was regarded as loud for participants of all ages. In the present study, >67% of maximum volume was loud for ≥18y, and >60% for participants <18y. In the present study, the mean age was 17.11 (95% CI: 16.72–17.50), and >60% was mostly applied as threshold of loud sound.
